# MRI analysis of the ISOBAR TTL internal fixation system for the dynamic fixation of intervertebral discs: a comparison with rigid internal fixation

**DOI:** 10.1186/1749-799X-9-43

**Published:** 2014-06-04

**Authors:** Jun Gao, Weihua Zhao, Xi Zhang, Luming Nong, Dong Zhou, Zhengxiang Lv, Yonghua Sheng, Xingbiao Wu

**Affiliations:** 1 Department of Orthopedics, Changzhou Traditional Chinese Medicine Hospital, Nanjing Traditional Chinese Medical University, Changzhou 213003, China; 2 Orthopedics Department, Changzhou No. 2 People's Hospital, Nanjing Medical University, Changzhou 213000, China

**Keywords:** Intervertebral disc degeneration, Magnetic resonance imaging, Dynamic fixation, Internal fixation

## Abstract

**Objectives:**

Using magnetic resonance imaging (MRI), we analyzed the efficacy of the posterior approach lumbar ISOBAR TTL internal fixation system for the dynamic fixation of intervertebral discs, with particular emphasis on its effects on degenerative intervertebral disc disease.

**Methods:**

We retrospectively compared the MRIs of 54 patients who had previously undergone either rigid internal fixation of the lumbar spine or ISOBAR TTL dynamic fixation for the treatment of lumbar spondylolisthesis. All patients had received preoperative and 6-, 12-, and 24-month postoperative MRI scans of the lumbar spine with acquisition of both routine and diffusion-weighted images (DWI). The upper-segment discs of the fusion were subjected to Pfirrmann grading, and the lumbar intervertebral discs in the DWI sagittal plane were manually drawn; the apparent diffusion coefficient (ADC) value was measured.

**Results:**

ADC values in the ISOBAR TTL dynamic fixation group measured at the 6-, 12-, and 24-month postoperative MRI studies were increased compared to the preoperative ADC values. The ADC values in the ISOBAR TTL dynamic fixation group at 24 months postoperatively were significantly different from the preoperative values (*P* < 0.05). At 24 months, the postoperative ADC values were significantly different between the rigid fixation group and the ISOBAR TTL dynamic fixation group (*P* < 0.05).

**Conclusion:**

MRI imaging findings indicated that the posterior approach lumbar ISOBAR TTL internal fixation system can prevent or delay the degeneration of intervertebral discs.

## Introduction

Lumbar fusion fixation is the main surgical method for the treatment of lumbar degenerative disease. However, it has been recently shown that rigid internal lumbar fixation has a tendency to cause adjacent segment degeneration, resulting in clinical symptoms and detectable imaging study abnormalities [1,2]. Because of these concerns, the dynamic fixation system has been employed in the fusion fixation procedure [3-5]. It has been suggested that intervertebral disc degeneration in the upper segments of the fused region is less with the dynamic fixation process. Between December 2008 and August 2011, we used the posterior approach lumbar ISOBAR TTL dynamic stabilization system to treat lumbar spondylolisthesis. Using pre and postoperative magnetic resonance images (MRIs), we studied the results of the ISOBAR system in comparison to traditional rigid internal fixation. We paid particular attention to the pre and postoperative MRI changes in the intervertebral discs.

## Materials and methods

### General materials

Between December 2008 and August 2011, 54 patients (19 men, 35 women) underwent surgery for the treatment of lumbar spondylolisthesis. The mean patient age was 59 years (range, 43–68 years), and symptoms had been present for 1–10 years. According to the Newman classification, there were 36 cases of isthmus spondylolysis and 18 cases of degenerative spondylolisthesis. This study was conducted in accordance with the declaration of Helsinki. This study was conducted with approval from the Ethics Committee of Nanjing Traditional Chinese Medical University. Written informed consent was obtained from all participants. Inclusion criteria were (1) lumbar spondylolisthesis patients with >2 years of follow-up and access to all clinical data and (2) patients who received conventional treatment for >0.5 year without any effects. The exclusion criteria were (1) severe osteoporosis, (2) local scoliosis or misalignment, and (3) traumatic lumbar spondylolisthesis, space occupying lesion, or bone fractures. Within the spondylolisthesis group, there were 4 cases of L3 spondylolisthesis, 31 cases of L4 spondylolisthesis, and 19 cases of L5 spondylolisthesis. According to the Meyerding grading, there were 6 cases of grade I, 31 cases of grade II, and 17 cases of grade III spondylolisthesis. Of the 54 patients, 24 (8 men, 16 women; age, 43–65 years; mean age, 58 years) were randomly assigned to the ISOBAR TTL dynamic fixation group, and 30 patients (11 men, 19 women; age, 51–65 years; mean age, 61 years) were assigned to the rigid internal fixation group. All patients had experienced low back pain and lumbar acid bilge feeling on the hips. Thirty-four patients had unilateral or bilateral sciatica, and 14 patients experienced intermittent claudication. Two patients with L4/L5 disc herniation had undergone discectomy 6 years and 2 years previously. All patients underwent lateral spine radiography in the entopic, double oblique flexion, and dynamic lateral positions. CT scans, routine MRI scans, and diffusion-weighted imaging (DWI) scans were performed in all patients. Results revealed 28 patients with lumbar spinal stenosis, and 35 patients with prolapse of a lumbar intervertebral disc. There were 9 patients with Pfirrmann grade I, 18 patients with grade II, 15 patients with grade III, 10 patients with grade IV, and 2 patients with grade V intervertebral disc degeneration in the upper segment of the fusion site.

The ISOBAR TTL dynamic stabilization system was obtained from General Scient'X (Arras, France). The dynamic stick included a controlled amphiarthrodial joint composed of overlapping internal Ti rings, allowing a ±2-mm longitude shift and ±2° 3D activity angle. The device acted to disperse the stress at the segments adjacent to the fusion and to maintain vertebral height and motion. The micromotion device of ISOBAR TTL was fixed on the rear of the adjacent facet joint.

The MRI scanning and acquisition of the DWI images employed SE sequence echo plain imaging technology (EPI) with sagittal view scanning. The parameters were TR, 2,300 ms; TE, 64.9 ms; matrix, 128 × 64; lay thickness, 4 mm; space interval, 1 mm; FOV, 34 cm; and scanning time, 37 s.

The gender, age, average follow-up time, preoperative Oswestry Disability Index (ODI) score, Department of Orthopedics of the Japan Society (JOA) score, and operation section of the two groups are shown in Table 1. There were no significant differences in gender, average age, and follow-up time between the two groups.

**Table 1:** The basic data of patients of the two groups

**Operation method**	**Gender (case)**		**Mean age (years)**	**Follow-up time (month)**	**Fusion segment**			
	**Male**	**Female**			**L**_**4**_**/L**_**5**_	**L**_**5**_**/S**_**1**_	**L**_**4**_**/S**_**1**_	**L**_**3**_**/L**_**5**_
ISOBAR TTL	8	16	58.3 ± 13.5	28.7 ± 5.3	4	5	8	7
PLIF	11	19	61.4 ± 15.2	30.1 ± 6.8	4	5	12	9

### Position and exposure

The patients were placed in the prone position. Using a posterior central straight incision of the lumbar spine, the spinous process, lamina, zygapophyseal joint, and vertebral pedicle were respectively exposed, and a pedicle screw was fixed.

### Decompression

Decompression laminectomy was performed bilaterally with resection of hyperplastic fibrous connective tissue, bone, and any thickened areas of the yellow ligament around the medial facet at the vertebral isthmus. The nerve root canal was smoothed, and the nucleus pulposus was removed to reduce pressure on the dural sac and nerve root.

### Implantation of internal fixation

**Figure 1:**
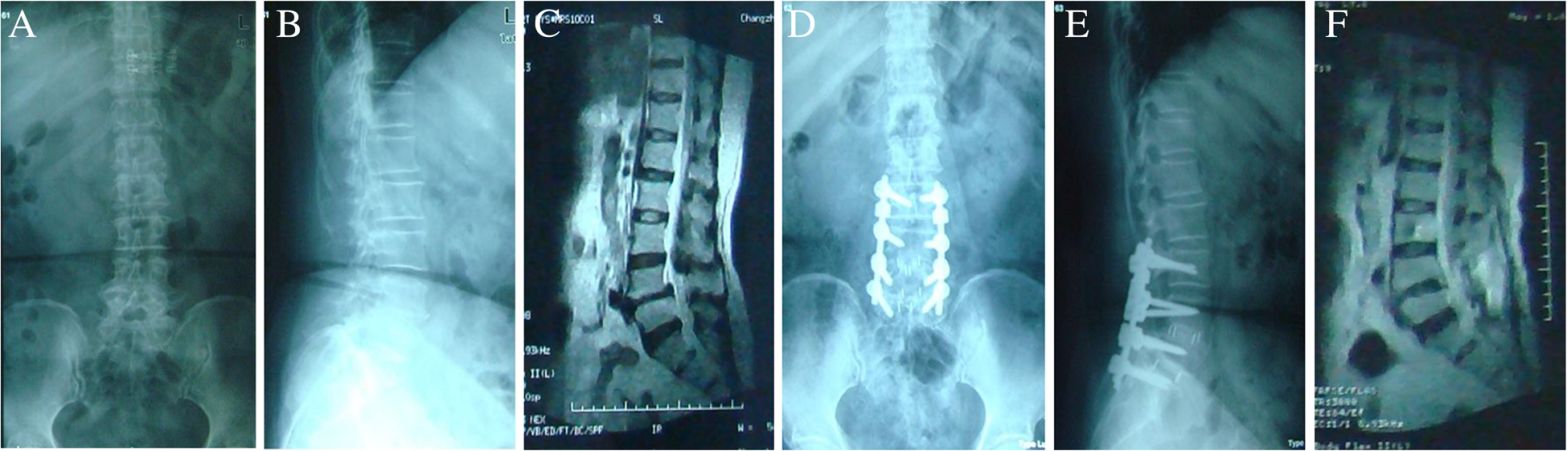
**Patient with L4/L5 spondylolisthesis I grade, L5/S1 and L4/L5 protrusion of intervertebral disc, spinal stenosis.** The patient is female and 64 years old. **(A)** Preoperative lumbar radiograph. **(B)** Preoperative lumbar lateral film. **(C)** MRI of the lumbar spine, intervertebral disc degeneration, L4/L5 intervertebral disc degeneration, V grade. **(D)** ISOBAR TTL internal fixation system, L3/L4 implanted microactuator device, the postoperative lumbar radiograph. **(E)** Postoperative lumbar lateral film. **(F)** Lumbar intervertebral disc MRI after 2 years, L3/L4 grade III degeneration, L4/L5 grade IV intervertebral disc degeneration.

**Figure 2:**
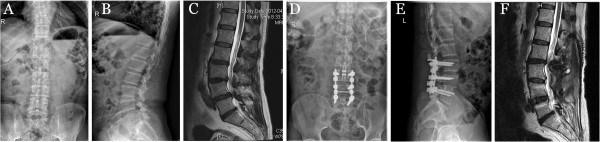
**Patient with L4/L5 grade I spondylolisthesis, L3/L4 and L4/L5 protrusion of intervertebral disc, spinal stenosis. (A)** Preoperative lumbar radiographs. **(B)** Preoperative lateral lumbar spine MRI. **(C)** L3/L4 grade IV intervertebral disc degeneration. **(D)** ISOBAR TTL internal fixation system, L3/L4 implanted microactuator device, the postoperative lumbar radiograph. **(E)** Postoperative lumbar lateral film. **(F)** Lumbar intervertebral disc MRI after 2 years, L3/L4 grade II lumbar intervertebral disc degeneration.

The intervertebral space was opened, and an intervertebral fusion cage was implanted at the lesion segment. Using autologous bone implantation and fusion, restoring of spondylolisthesis, and connecting rod, the micromotion device of the ISOBAR TTL group was fixed to the rear of the adjacent facet joint (Figure 1D,E and Figure 2D,E).

### Postoperative treatment and rehabilitation

Negative pressure drainage and antibiotics to prevent infection were routinely used postoperatively. Ambulation with a brace was permitted 1–2 weeks postoperatively.

### Clinical therapeutic evaluation

The ODI score and lumbocrural pain curative standard (29-point method) enacted by the JOA in 1984 were used to measure dysfunction during the preoperative and postoperative periods.

### MRI analysis

The ISOBAR TTL dynamic fixation group and rigid internal fixation group both underwent MRI preoperatively and at 6, 12, and 24 months postoperatively to evaluate the intervertebral discs of the upper segment of fusion and fixation (Figures 1F and 2F).

### Lumbar intervertebral disc Pfirrmann grading

Pfirrmann intervertebral disc degeneration grading standards [6] are based on sagittal T2-weighted images of the nucleus pulposus signal changes. The intervertebral disc is divided into five levels according to the boundaries of the nucleus pulposus and annulus. The MRI images were independently evaluated by an experienced radiologist and an orthopedic surgeon. The intervertebral discs were graded according to the Pfirrmann evaluation standard.

### Setting and measurement of the region of interest

The nucleus pulposus in the upper segment of the fusion and fixation was drawn by a trained, experienced radiologist, with the median sagittal plane DWI as the measuring plane. This was defined as the region of interest (ROI). The following grading was used: (1) I–II grade: an ellipse ROI in center part of the disc was drawn, containing the nucleus, about the size of 60–80 mm^2^, (2) III–V grade: because the boundary of nucleus pulposus in fiber ring was not clear, irregular or oval ROI could be drawn, according to the position of the nucleus pulposus, (3) every ROI measurement was repeated three times, taking the mean value and recording every disc ROI and apparent diffusion coefficient (ADC) [7].

### Statistical analysis

All imaging data were reviewed by an experienced radiologist and an orthopedic surgeon in an independent and double blind manner. The data were subjected to statistical analysis using SPSS13.0 statistical software. The results of the ODI scores, JOA scores, and ADC values were compared using the paired comparison methods, and significant differences were defined as *P* < 0.05.

## Results

Fifty-four cases were followed up for a period of 24–48 months. Lumbar lateral radiographs and the lumbar MRIs from 6, 12, and 24 months postoperatively were reviewed.

### Clinical curative evaluation

The JOA and ODI scores of the two groups of patients were significantly improved at 6-, 12-, and 24-month postoperative evaluations compared with the preoperative scores (*P* < 0.05). The therapeutic effect between the two groups was not significantly different (*P* > 0.05) (Tables 2 and 3).

**Table 2:** **Comparison of preoperative and postoperative JOA total scores of the two groups of patients **$\left(\overset{¯}{x}\pm s\right)$

**JOA**	**Preoperative score**	**Score 6 months after operation**	**Score 12 months after operation**	**Score 24 months after operation**
ISOBAR TTL	9.45 ± 2.19	18.75 ± 2.56*	22.82 ± 3.07*	26.17 ± 3.67*
PLIF	10.94 ± 2.47	16.33 ± 1.86*	20.75 ± 2.56*	22.46 ± 3.97*
*P*	>0.05	>0.05	>0.05	>0.05

Compared with preoperative, *P < 0.05.

**Table 3:** **Comparison of preoperative and postoperative ODI values of the two groups of patients **$\left(\overset{¯}{x}\pm s\right)$

**ODI**	**Preoperative score**	**Score 6 months after operation**	**Score 12 months after operation**	**Score 24 months after operation**
ISOBAR TTL	60.36 ± 11.25	20.18 ± 6.46*	15.07 ± 4.82*	11.83 ± 5.68*
PLIF	58.19 ± 10.83	22.16 ± 6.17*	18.43 ± 5.66*	13.28 ± 3.37*
*P*	>0.05	>0.05	>0.05	>0.05

Compared with preoperative, *P < 0.05.

### MRI imaging evaluation

Using the Pfirrmann grade standard of intervertebral disc degeneration, the preoperative and postoperative findings were compared. In the ISOBAR TTL group after 24 months, two cases of grade II and 1 case of grade III degenerative intervertebral discs improved to grade I. Additionally, five cases of grade III degeneration improved to grade I. There were four cases of grade IV degeneration that improved to grade II in two patients and grade III in two patients. One case of grade V degeneration improved to grade III, while another case of grade V improved to grade VI. One case of grade II intervertebral disc degeneration and 1 case of grade III degeneration improved to grade III and IV, respectively.

In the rigid internal fixation group at 24 months postoperatively, of the eight cases with preoperative grade I intervertebral disc degeneration, five worsened to grade II and three to grade III. Of the 12 cases with preoperative grade II degeneration, 8 worsened to grade III and 4 to grade VI. The three cases of grade III intervertebral disc degeneration worsened to grade IV in two cases and grade V in one case. In the rigid internal fixation group at three follow-up time quantum, the ADC value was decreased compared to the preoperative value. The ADC value at 24 months postoperatively significantly decreased compared to the preoperative value (*P* < 0.05). The 6- and 12-month ADC values were not significantly different from the preoperative values (*P* > 0.05), but at 24 months postoperatively, the ADC value was significantly different (*P* < 0.05).

The ADC values of the intervertebral discs preoperatively and at 6, 12, and 24 months postoperatively are shown in Table 4. One case in the ISOBAR TTL group displayed an artifact which precluded measurement of the ADC value, and this case was excluded from the ADC analysis. Compared with the preoperative values, the ADC values of the ISOBAR TTL group increased at each follow-up evaluation and reached a level of statistical significance at the 24-month evaluation (*P* < 0.05). The ADC values of the rigid internal fixation group decreased at the 3-month follow-up compared to the preoperative values. The ADC values were significantly different at 24 months postoperatively (*P* < 0.05). The ADC values showed no statistically significant difference between the two groups before and at 6 and 12 months postoperatively (*P* > 0.05), but at 24 months postoperatively, there was a significant difference in the ADC values between the two groups (*P* < 0.05).

**Table 4:** **Comparison of preoperative and postoperative ADC values of the two groups of patients **$\left(\overset{¯}{x}\pm s,\times 10^{-3}\;{\mathrm{mm}}^{2}/s\right)$

**T2 value**	**Preoperative score**	**Score 6 months after operation**	**Score 12 months after operation**	**Score 24 months after operation**
ISOBAR TTL	1.03 ± 0.28	1.13 ± 0.31	1.11 ± 0.37	1.25 ± 0.24*
PLIF	1.16 ± 0.40	1.08 ± 0.22	1.01 ± 0.25	0.94 ± 0.28*
*P*	>0.05	>0.05	>0.05	<0.05

Compared with preoperative, *P < 0.05.

## Discussion

The increase of adjacent segment compensatory activity after rigid internal fixation is one of the most important causes of adjacent segment degeneration [5]. This disadvantage of the fixed rigid internal fixation system has led to the concept of dynamic lumbar fixation. ISOBAR TTL is a type of semi-rigid lumbar, posterior approach, dynamic, and screw rod fixation system. This system allows some mobility of the fixation segments, maintains height of intervertebral space, reduces the bearing load of the discs and facet joints, and further prevents or slows down the degeneration of the fixation segment. The ISOBAR TTL internal fixation system can share 43% of the stress on the dynamic fixed segment disc, which can prevent intervertebral disc degeneration [8]. Through the analysis of the finite element simulation model, some researchers have found that there was no significant difference in the lower lumbar range of motion between ISOBAR TTL internal fixation system and the normal model. This indicates that the ISOBAR TTL system can effectively maintain postoperative lumbar activity and reduce stress shielding, which could theoretically slow the dynamic fixed segment degeneration [9].

Most current standards of lumbar disc degeneration include a combination of many factors, including histological manifestations, radiographs, and grade, allowing one to estimate the degree of disc degeneration with consistency [10]. Intervertebral disc degeneration occurs when the nucleus pulposus dehydrates, the fiber ring fractures, and the intervertebral space collapses. Dehydration of the nucleus pulposus is the initial step in intervertebral disc degeneration. The moisture content of the nucleus pulposus is about 90% in a newborn baby, and about 80% in an adult. MRI is the most sensitive method for detecting the degeneration of the lumbar nucleus pulposus and annulus fibrosus. The loss of moisture, protein, and polysaccharides in the nucleus pulposus with aging leads to the degeneration of the intervertebral disc. The MRI T2 signal changes with this degeneration, and it has been shown that the level of T2 signal significantly correlates to the severity of degeneration in the lumbar spine [11].

Based on the MRI assessment of lumbar disc degeneration, the Pfirrmann system integrates four quantitative indicators: the level of nucleus pulposus signal, the nucleus pulposus distribution, the boundary of the nucleus pulposus, and the fiber content and the height of the intervertebral disc. This MRI classification is recognized as the main standard in determining the degree of lumbar disc degeneration [6,12,13]. Cuellar et al. [14] compared the Pfirrmann score in 28 cases of failed nucleoplasty patients before and after intervertebral degenerative disc surgery and found that the scores of about 32% of the patients increased significantly 1 year after the operation. These results are consistent with previous theories suggesting accelerated adjacent disc degeneration after nucleoplasty. This study confirmed the feasibility of using the Pfirrmann system clinically and prognostically to evaluate intervertebral disc degeneration in the lumbar spine. In our study, at 24 months postoperatively, intervertebral disc degeneration in the ISOBAR TTL group slowed, and 14 dynamic fixation intervertebral discs showed improvement in Pfirrmann grade. However, in the rigid fixation group, there were 23 discs which showed higher grade degeneration at 24 months postoperatively.

DWI is currently the only non-invasive technology to measure *in vivo* water molecule diffusion, which can reflect the structural characteristics of tissue. Research shows that the pathogenesis of intervertebral disc degeneration may be associated with a decrease of the diffusion rate [15,16]. Based on quantitative studies of diffusion of living tissue, DWI may have potential value in the early diagnosis of intervertebral disc degeneration [17,18]. DWI may also allow the non-invasive evaluation of therapeutic efficacy and provide prognostic information in these patients. In DWI, the higher the intervertebral disc water content, the faster the molecular diffusion and the higher the ADC value, reflecting a healthier nucleus pulposus. The lower the intervertebral disc water content, the slower the molecular diffusion, and the lower the ADC value indicate a higher degree of degeneration of the intervertebral disc. The purpose of this study was the quantitative evaluation of intervertebral disc integrity by evaluating the DWI of the fixed adjacent intervertebral disc. Compared with the preoperative studies, at the 6- and 12-month postoperative evaluations, neither group demonstrated significant changes in ADC values (*P* < 0.05). However, at 24 months postoperatively, the ADC value was significantly different (*P* < 0.05). The ADC values in the ISOBAR TTL group at 6, 12, and 24 months after surgery were higher than those before surgery, which showed that the nucleus pulposus water content postoperatively was increased and that the degree of intervertebral disc degeneration could be slowed or even reversed. In the rigid fixation group, the ADC values were lower than the preoperative value at the 6-, 12-, and 24-month evaluations, indicating that the nucleus pulposus water content of the disc decreased after surgery and that the degree of intervertebral disc degeneration worsened. MRI images indicated that the posterior approach lumbar ISOBAR TTL internal fixation system can prevent or delay intervertebral disc degeneration.

## Conclusion

The posterior approach lumbar ISOBAR TTL internal fixation system can prevent or delay the degeneration of dynamic fixation intervertebral discs. Previously, few imaging studies have evaluated the effect of the ISOBAR TTL internal fixation system on the degeneration of dynamic fixation intervertebral discs. Larger, comprehensive, and long-term randomized studies that evaluate the efficacy of this internal fixation system are necessary.

## Competing interests

The authors declare that they have no competing interests.

## Authors' contributions

JG and WZ participated in the design of the study and drafted the manuscript. XZ, ZL, LN, and DZ participated in the design and coordination of the study and helped draft the manuscript. YS and XW participated in the design of the study and performed the statistical analysis. All authors read and approved the final manuscript.
